# The right to live or die? A perspective on voluntary euthanasia

**DOI:** 10.12669/pjms.305.5777

**Published:** 2014

**Authors:** Amber Shah, Ammara Mushtaq

**Affiliations:** 1 Amber Shah, Final Year Medical Student, Sind Medical College, Dow University of Health Sciences, Karachi, Pakistan.; 2 Ammara Mushtaq, Final Year Medical Student, Dow Medical College, Dow University of Health Sciences, Karachi, Pakistan.

**Keywords:** Euthanasia, Physician-assisted death

## Abstract

“It is choice alone that is being honored, without regards for what is chosen.” The debate on euthanasia in medical community stays unresolved. In this manuscript, we present arguments for and against euthanasia, review arguments from both the sides and conclude it with our opinion.

In the medical community, the debate over euthanasia has been going since ages. However, there have been no concrete conclusions to answer the imperative question: do we have a right to assess whether a life is worth living?

The Expert Panel on End of Life Decision-Making established by the Royal Society of Canada defines voluntary euthanasia as “an act undertaken by one person to kill another person whose life is no longer worth living to them in accordance with the wishes of that person”^1^ and this definition is operative throughout our discussion.

To decide to end a life can be an extremely complicated decision. As the cliché goes, it’s easier said than done. There can be many reasons cited for ending a life: The patient can be in unbearable pain, in vegetative state for a long time, or a terminally ill patient suffering from an uncurable disease. Regardless of the reason, should euthanasia be legalized?

A study was done by Washington State on the most frequent reasons cited by patients who opted for euthanasia. Amongst the top of the list were loss of control, independency, and dignity; and being a burden on others for personal care.^2^ Robert Pearlman, a physician specializing in geriatric care, did a study on patient’s outlooks on states worse than death and showed that 96% felt it was better to die than to be kept alive under hopeless circumstances with impending death.^3^

A study by the American Psychological Association amongst cancer patients on their views on euthanasia showed that 62.8% patients contemplated that euthanasia should be legalized, and 39.8% would consider making a future request for a physician-hastened death. The desires to receive euthanasia amongst the patients receiving palliative care for cancer were connected with religious beliefs; functional status; and physical, social, and psychological symptoms and concerns.^4^

Pasman et al. conducted a study on what patients and physicians considered to represent unbearable suffering. Patients gave more significance to psychosocial suffering, such as dependence and loss of autonomy while physicians put more emphasis on physical suffering.^5^

An earlier paper reported the number of requests occurring in Dutch general practice between 1977 and 2001. The main finding was that after a steep rise during the first 20 years of registration, the request for euthanasia mainly by cancer patients became steady. Further scrutiny led to the conclusion that hopelessness and deterioration were relatively constant reasons for a request, whereas pain and dyspnoea were gradually decreasing reasons.^6^

Euthanasia was legalized in Belgium in 2002 for adults under strict conditions and is legal in at least 6 other jurisdictions: the Netherlands, Luxemburg, Switzerland, Oregon, Washington State, and Montana.^1^ The rules and conditions were that patient must be in a medically futile condition and must be in constant and unbearable physical or mental suffering that cannot be alleviated, which has resulted from a serious and incurable disorder caused by illness or accident.^7^ The frequency of reported euthanasia cases has increased every year since legalization.^7^ Those who died from euthanasia (compared with other deaths) were more often younger, male, cancer patients and more often died in their homes. In almost all cases, unbearable physical suffering was reported.^7^

Many arguments have been advocated against euthanasia. First one is that life being sacred, should be preserved. Theists believe that God gives us life and He is the one who decides our death. Opponents also argue that when a physician takes Hippocrate oath, his first duty is to save life. They contend that euthanasia devalues life. More importantly, they argue that assisting death to the terminally ill patients will lead to a “slippery slope” in which euthanasia is extended to non-terminally ill patients and those who don’t ask for it. Families and loved ones might prioritize financial gains over saving a life. Despite this, the public support for decriminilization of euthanasia remains high.^1^

An example of misuse of euthanasia is the case of Dr. Jack Kevorkian, who used unethical methods of offering euthanasia to many who request it regardless of the condition. Some of his patients were not terminally ill and could have committed suicide without assistance if they wanted to. Dr. Kevorkian didn’t follow proper medical regulations and procedures while donating his patient’s organs for transplantation. This has caused widespread fear and abhorrence of euthanasia.

Assisting death does not imply that the patient should not receive the most palliative medical care possible. It rather means being compassionate towards the patient’s desires and making death with dignity a real option for him. “The p-word is not 'pain’; the p-word is 'pride',” Oregon oncologist Kenneth Stevens told the New York Times. "Rather than being death with dignity, it's death with vanity."

Euthanasia is a very controversial topic and will always lead to passionate discussions but its vital for authorities to design elaborate guidelines to optimize end-of-life practices.

## Authors’ Contributions:

**AS:** Manuscript drafting, data collection.

**AM:** Conception of idea, critical revision and editing.

## Notes

***Financial interest:*** None.
